# Cell Arrest and Cell Death in Mammalian Preimplantation Development: Lessons from the Bovine Model

**DOI:** 10.1371/journal.pone.0022121

**Published:** 2011-07-21

**Authors:** Sandra Leidenfrost, Marc Boelhauve, Myriam Reichenbach, Tuna Güngör, Horst-Dieter Reichenbach, Fred Sinowatz, Eckhard Wolf, Felix A. Habermann

**Affiliations:** 1 Institute of Anatomy, Histology and Embryology, Department of Veterinary Sciences, Ludwig-Maximilians-University Munich, Munich, Germany; 2 Chair for Molecular Animal Breeding and Biotechnology, Department of Veterinary Sciences, and Laboratory for Functional Genome Analysis (LAFUGA), Gene Center, Ludwig-Maximilians-University Munich, Munich, Germany; 3 Biotechnology Unit, Institute of Animal Breeding, Bavarian State Institute for Agriculture, Poing, Germany; 4 South Westphalia University of Applied Sciences, Soest, Germany; University of Muenster, Germany

## Abstract

**Background:**

The causes, modes, biological role and prospective significance of cell death in preimplantation development in humans and other mammals are still poorly understood. Early bovine embryos represent a very attractive experimental model for the investigation of this fundamental and important issue.

**Methods and Findings:**

To obtain reference data on the temporal and spatial occurrence of cell death in early bovine embryogenesis, three-dimensionally preserved embryos of different ages and stages of development up to hatched blastocysts were examined *in toto* by confocal laser scanning microscopy. In parallel, transcript abundance profiles for selected apoptosis-related genes were analyzed by real-time reverse transcriptase-polymerase chain reaction. Our study documents that *in vitro* as well as *in vivo*, the first four cleavage cycles are prone to a high failure rate including different types of permanent cell cycle arrest and subsequent non-apoptotic blastomere death. *In vitro* produced and *in vivo* derived blastocysts showed a significant incidence of cell death in the inner cell mass (ICM), but only in part with morphological features of apoptosis. Importantly, transcripts for CASP3, CASP9, CASP8 and FAS/FASLG were not detectable or found at very low abundances.

**Conclusions:**

*In vitro* and *in vivo*, errors and failures of the first and the next three cleavage divisions frequently cause immediate embryo death or lead to aberrant subsequent development, and are the main source of developmental heterogeneity. A substantial occurrence of cell death in the ICM even in fast developing blastocysts strongly suggests a regular developmentally controlled elimination of cells, while the nature and mechanisms of ICM cell death are unclear. Morphological findings as well as transcript levels measured for important apoptosis-related genes are in conflict with the view that classical caspase-mediated apoptosis is the major cause of cell death in early bovine development.

## Introduction

Bovine preimplantation embryos represent an attractive model for investigating fundamental mechanisms of early mammalian development [1]. In important aspects, e.g. the timing of epigenetic reprogramming and embryonic genome activation, bovine embryos reflect far more closely the situation in human embryos and in other non-rodent mammals than the mouse model [2]. The bovine model is valuable for elucidating the mechanisms of folliculogenesis, oocyte maturation and reproductive ageing in women. Important similarities to humans include the emergence of follicular waves, the number of waves during the menstrual/estrous cycle, selection of a dominant follicle and ovulation of a single follicle [3].

As in other mammalian species, early embryo death before implantation is a major determinant of fertility in cows [4]. While estimates for the fertilization rate in dairy cows after artificial insemination (AI) range between 80 and 100 percent, recently published conception rates range around 45 percent [5], [6].

*In vitro* and *in vivo*, the development of mammalian zygotes to the blastocyst stage is highly variable and the individual outcome is uncertain. Cell death during this process has been reported, with ambiguous interpretations ranging from a pathological phenomenon to an indispensable part of normal blastocyst development [4]. Current knowledge on the occurrence of cell death in early bovine embryos is largely based on epifluorescence microscopy studies: namely on the morphological analysis of cell nuclei stained with DNA-specific dyes such as DAPI and on the detection of DNA fragmentation using TUNEL (terminal deoxynucleotidyl transferase-mediated dUTP nick end labeling). Nuclear chromatin condensation and nuclear fragmentation as well as DNA fragmentation are classical hallmarks of programmed cell death (PCD)/apoptosis. Depending on the species, such signs of PCD/apoptosis have been described already in early cleavage stage embryos [7]. Apoptosis as defined by DNA-staining and the TUNEL assay has been described as a frequent finding in blastocysts from cattle, pigs, humans and other mammals [7]. In a study of bovine blastocysts produced *in vitro* or *in vivo*, cells with morphological signs of apoptosis were described in practically all embryos, predominantly in the inner cell mass (ICM) [8]. Transmission electron microscopy (TEM) of blastocysts seemed to provide further pieces of evidence for apoptosis especially in the ICM: cells with highly condensed cytoplasm, nuclei with condensed and marginalized chromatin, fragmented nuclei as well as apoptotic bodies containing nuclear fragments and intact organelles [9]. Hardy et al. [10] reported high apoptotic indices in morphologically excellent human blastocysts produced *in vitro* and proposed that programmed cell death might play an important role in normal development, e.g., by regulating the cell number. Studies in cattle and other mammalian species determined higher apoptotic indices in blastocysts produced *in vitro* compared to those developed *in vivo* [7], [11], concluding that insufficient *in vitro* embryo production systems might increase apoptosis. Accordingly, the incidence of apoptosis was proposed as an indicator of the health status and developmental potential of morulae and blastocysts.

**Figure 1:**
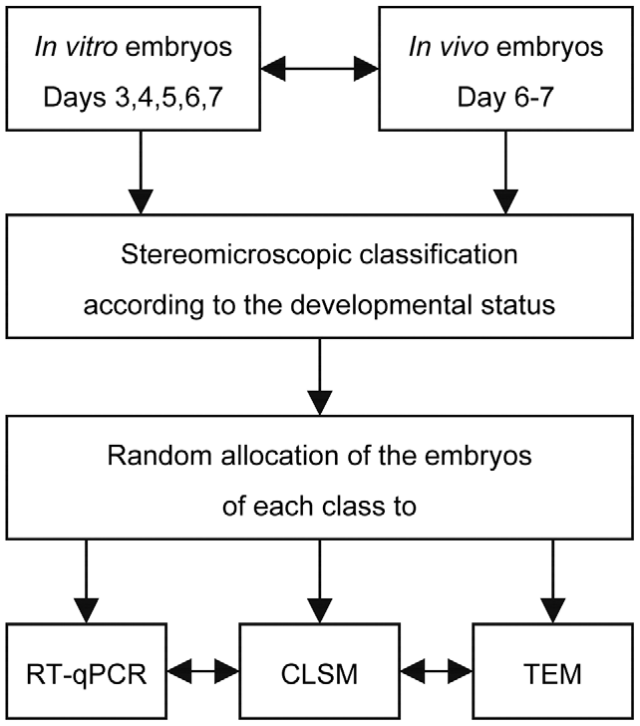
Experimental design for the analysis of cell arrest and cell death in early bovine embryos.

Despite a number of previous studies on this topic, the time course and nature of cell death during mammalian preimplantation development is only incompletely resolved. Therefore, we performed a comprehensive study of early bovine embryogenesis from fertilization to the hatching blastocyst. In particular, we addressed the increase in the cell number and the occurrence and nature of cell death by 3D confocal laser scanning microscopy (3D CLSM). Further, classical cell death pathways were screened by quantitative real-time PCR (RT-qPCR) analysis of transcript copy numbers of ten selected genes which have been shown to play decisive roles in the execution, initiation and regulation of apoptosis. The study design is shown in Figure 1.

## Materials and Methods

All animal and *in vitro* procedures were conducted according to the German Animal Welfare Act (Tierschutzgesetz). Bull semen was donated by Bayern Genetik GmbH, Grub, Germany. Estrous cow serum was donated by BFZF GmbH, Oberschleißheim, Germany. Bovine ovaries were obtained from a slaughterhouse (Münchner Schlachthof Betriebs GmbH, Munich, Germany). Both *in vitro* and *in vivo* produced embryos were obtained from an EU approved bovine embryo collection and production centre at the Chair for Molecular Animal Breeding and Biotechnology of the LMU Munich (Moorversuchsgut Badersfeld, Oberschleißheim, Germany; approval number for the *in vivo* and *in vitro* procedures issued by the District Government of Upper Bavaria, Munich, Germany: DE ETR 006 EWG).

### *In vitro* production of bovine embryos

The standardized protocol for *in vitro* production (IVP) of bovine embryos was described previously [12]. Briefly, ovaries from slaughtered cows (predominantly Simmental Fleckvieh) were washed several times with phosphate buffered saline (PBS). Cumulus-oocyte complexes (COCs) were aspirated from 3–8 mm follicles and classified using a stereomicroscope. Only COCs with homogenous cytoplasm and surrounded by at least three compact layers of cumulus cells (IETS grade 1 and 2 [13]) were chosen for the experiments and washed twice in oocyte maturation medium (TCM-199 with Earle's salts, supplemented with 5% estrous cow serum, 0.025 IU/ml FSH and 0.0125 IU/ml LH). Groups of 30 40 COCs were matured *in vitro* (IVM) for 22 h in 400 µl oocyte maturation medium at 39°C in an atmosphere of 5% CO_2_ in humidified air. After IVM, COCs were washed in IVF-TALP (modified Tyrode's solution supplemented with 6 mg/ml BSA, 0.022 mg/ml pyruvic acid, and 0.01 mg/ml heparin) and transferred to 4-well plates that contained 400 µl of IVF-TALP per well.

For all *in vitro* fertilization (IVF) experiments frozen semen from the same Simmental Fleckvieh bull was used. Motile spermatozoa were selected by swim-up in Sperm-TALP (modified Tyrode's solution supplemented with 6 mg/ml BSA and 0.11 mg/ml pyruvic acid) at 39°C in an atmosphere of 5% CO_2_ in humidified air. Approximately 18 h after addition of spermatozoa suspension (∼1×10^6^ spermatozoa/ml), the cumulus cells were removed by gentle vortexing for 3 min. Groups of 30–40 presumptive zygotes were transferred to 400-µl drops of synthetic oviduct fluid (SOF) supplemented with 5% (v/v) estrous cow serum, essential and non-essential amino acids, covered with mineral oil and cultured at 39°C in a humidified atmosphere of 5% CO_2_, 5% O_2_ and 90% N_2_. In all experiments, the same batch of estrous cow serum was used. Embryos were harvested for analysis on day 3 (72 h), 4 (96 h), 5 (120 h), 6 (144 h) and 7 (168 h) after sperm addition.

### *In vivo* production of bovine embryos

Ten 15- to 21-month-old Fleckvieh heifers ranging from 330 to 395 kg body weight were used twice at an interval of seven weeks in two separate experiments to produce embryos *in vivo* by superovulation (SO) and artificial insemination (AI). The heifers were hormonally synchronized by using a progesterone-releasing intravaginal device (PRID; Ceva, Düsseldorf, Germany) containing 1.55 g progesterone plus 10 mg estradiol. Starting four days after PRID insertion, the animals received eight intramuscular injections of a 1∶1 mixture of FSH and LH (Pluset; Laboratorios Callier, Barcelona, Spain) at 12-h intervals in decreasing doses (100, 75, 75, 50, 50, 25, 25, 25 IU of each hormone). At the sixth and seventh injection, the animals additionally received two intramuscular doses of 500 µg of the synthetic prostaglandin F_2α_ (PGF_2α_) Cloprostenol (Estrumate; Essex, Munich, Germany). Seven days after insertion, at the seventh hormone injection, the PRID was removed. 22, 37 and 46 h after the eighth FSH/LH-injection, the heifers were artificially inseminated three times with frozen-thawed semen from the same bull used for the IVF experiments. At the first AI, the animals received a single intramuscular dose of 20 µg of the synthetic gonadotropin releasing hormone (GnRH) Buserelin (Receptal; Intervet, Unterschleissheim, Germany). On day 7 (∼160 h after the third AI), embryos were collected non-surgically by flushing each uterine horn with 250 ml pre-warmed (37°C) PBS.

### Stereomicroscopic classification and sorting of bovine embryos

Immediately after harvesting, the embryos were analyzed under a stereomicroscope equipped with a temperature-controlled heated stage at 80× magnification and classified and sorted according to their developmental stage by an experienced examiner. Unfertilized and uncleaved oocytes as well as severely retarded and degenerated embryos were not further analyzed. The *in vivo* produced embryos were classified and sorted according to IETS recommendations [14]. Only grade 1 and 2 blastocysts were selected for further analyses. The embryos were randomly allocated to CLSM, TEM and RT-qPCR analyses.

### Whole-mount preparation of bovine oocytes and embryos and confocal laser scanning microscopy (CLSM)

After a brief wash with PBS/PVP the embryos were fixed with 1.3% (w/v) paraformaldehyde in PBS for 60 min, followed by washing with PBS containing 1 mg/ml polyvinyl pyrrolidone (PVP). For the morphological analysis of cell nuclei and mitotic figures and the evaluation of cell death, DNA was stained with DAPI. In addition, DNA fragmentation was visualized *in situ* by terminal transferase-mediated dUTP nick end labeling (TUNEL) [15] and fluorescein-dUTP using a commercial kit (Roche, Mannheim, Germany) following manufacturer's instructions. As a positive control, the specimen were incubated in 50 IU/ml DNase I (Sigma, Taufkirchen, Germany) for 30 min at 37°C before the TUNEL reaction. As a negative control, terminal transferase was omitted from the reaction mixture. After completion of the TUNEL assay, the embryos were mounted in Vectashield antifade solution with DAPI (Axxora, Lörrach, Germany) on coverslips in such a way that the three-dimensional structure of the specimen was maintained. A series of embryos was stained with phalloidin-TRITC to visualize the f-actin cytoskeleton. Stacks of optical serial sections (optical thickness 1 µm) were recorded using a confocal laser scanning microscope (LSM 510 Meta, Zeiss) with a 40× PlanNeofluar (NA 1.3) oil immersion objective. For excitation of DAPI, fluorescein and TRITC laser lines of 364 nm, 488 nm and 543 nm were used. The resulting fluorescence emissions were detected through emission bandpass filters at 385–470 nm (Hoechst 33342), 505–530 nm (TUNEL) and 560–615 nm (Phalloidin-TRITC). The standard pixel size was 350×350 nm; the axial distance between optical sections was 1 µm. At each section plane, the signals of the three fluorochromes were sequentially recorded and saved as 8-bit grayscale images. Selected embryos were scanned with a smaller pixel size of 125×125 nm.

The confocal image stacks were analyzed with ImageJ software (National Institute of Health, Bethesda, MD, USA). For each embryo we determined a) the total number of cell nuclei and mitotic figures and b) the number of decaying (dying/dead) cells (condensed, fragmented and degraded nuclei as well as residues of mitotic figures) by comparing each optical serial section with the adjacent section. Specifically, we analyzed the correlation between cell death as evidenced by DAPI staining and DNA fragmentation as detected by the TUNEL assay.

### Quantitative real-time RT-PCR analysis

Amplification primers (see Table 1) were designed using Primer Express software version 2.0 (Applied Biosystems, Darmstadt, Germany) based on public transcript and genomic sequence data from cattle, mice and humans. For each gene analyzed, a transcript-specific plasmid DNA standard was generated as described previously [12]. The plasmid copy number was calculated from the molecular mass of the plasmid and the insert. The concentration of the plasmid standard solutions was adjusted to 10^6^ copies/µl.

**Table 1:** Transcripts and primers for quantitative real-time RT-PCR analysis.

Transcript		Primer sequence (5′-3′)	GenBank accession number	Fragment size (bp)
*18S rRNA*	sense	AAA CGG CTA CCA CAT CCA AGG	DQ066896	138
	antisense	GCG GAA GGA TTT AAA GTG GAC TC		
*H2AFZ*	sense	CGA AAT GGC TGG CGG TAA G	NM_174809	134
	antisense	GGC TAG TCG TCC TAG ATT TCA GGT		
*CASP3*	sense	CTG GAA AAC CCA AAC TTT TCA TTA	AY575000	165
	antisense	GCC AGG AAA AGT AAC CAG GTG C		
*BIRC4*	sense	GAA GCA CGG ATC ATT ACA TTT GG	AF458770	89
	antisense	CCT TCA CCT AAA GCA TAA AAT CCA		
*BAX*	sense	GCA GAG GAT GAT CGC AGC TG	U92569	197
	antisense	CCA ATG TCC AGC CCA TGA TG		
*BCL2*	sense	CTT CGC CGA GAT GTC CAG TC	AF515848	96
	antisense	CAC CAC CGT GGC GAA GC		
*BCL2L1*	sense	CGT GGA AAG CGT AGA CAA GGA G	AB238936	133
	antisense	GTA GAG TTC CAC AAA AGT GTC		
*CASP9*	sense	CGA CGC TTC CAC CTG CTG	NM_032996	219
	antisense	CAC AAT TCT CTC GAC GGA CAC AG		
*STAT3*	sense	CTG CAG CAG AAG GTT AGC TAC AAA	AJ620655	85
	antisense	TTC TAA ACA GCT CCA CGA TTC TCT		
*CASP8*	sense	GGC CAT GTC AGA CTC TCC AGA AC	DQ319070	204
	antisense	CGA AAG GTC TTA TCC AAA GCG TC		
*FAS*	sense	GCA ACT CTG CAG CCT CAA ATG	U34794	153
	antisense	CAT CTA TTT TGG CTT CTT CCA TAC C		
*FASLG*	sense	TCC ACC AGC CAA AGG CAT AC	AB035802	126
	antisense	GAT GGA TCT TGA GTTGAG CTT GC		

RNA isolation and reverse transcription was performed as described previously [12], [16] with minor modifications. Pools of ten *in vitro* matured oocytes, of ten *in vitro* embryos or of five *in vivo* embryos were snap-frozen and stored at −80°C. From each pool, total RNA was isolated by guanidine-thiocyanate/phenol/chloroform extraction [17]. To eliminate any contaminating DNA, a DNase digestion step was performed. The total RNA of each pool was reverse transcribed into cDNA in a reaction volume of 50 µl. The reaction mixture contained 0.15 µg/µl random hexamer primers (Invitrogen), 0.5 mM of each dNTP, 20 mM dithiothreitol (DTT, Invitrogen) and 2 IU/µl RNase inhibitor (RNaseOUT, Invitrogen), 1× transcription buffer and 1.2 IU/µl Superscript II reverse transcriptase (Invitrogen). Reverse transcription was allowed to proceed for 120 min at 42°C and terminated by heating to 70°C for 15 min.

To evaluate the efficiency and reproducibility of RNA extraction and reverse transcription we used pooled samples of *in vitro* matured oocytes and rabbit HBA mRNA (GenBank accession ID X04751) as an external control mRNA. To amplify a 145 bp fragment of rabbit HBA cDNA the following primers were used: sense - 5′ GCA GCC ACG GTG GCG AGT AT 3′ and antisense - 5′ CAG GGC TTC GGA CAC CTT C 3′. To generate a qPCR calibration curve for the control mRNA a plasmid DNA standard was prepared as described above.

All RT-qPCR analyses were performed in triplicate using an ABI PRISM 7000 sequence detector system (Applied Biosystems, Darmstadt, Germany) and SYBR Green as fluorescent reporter for double-stranded DNA. SYBR Green I and ROX reference dye were purchased from Invitrogen (Karlsruhe, Germany). HotFireTaq polymerase and 10× Taq reaction buffer B, dNTPs and MgCl_2_ were obtained from Solis Biodyne (Tartu, Estonia). A 25 µl qPCR reaction contained 1 µl cDNA sample solution corresponding to 0.1 oocyte or embryo equivalent, a final dilution of 1∶500,000 of SYBR Green I stock solution, 0.5 µM ROX reference dye, 1× Taq reaction buffer B, 200 µM of each dNTP, 4 mM MgCl_2_, 0.01 IU/µl uracil N-glycosylase, 0.3 µM of each primer and 0.04 IU/µl HotFire Taq polymerase. The cycling profile was: uracil N-glycosylase activation for 5 min at 50°C; initial denaturation for 15 min at 95°C; 40 cycles of 15 sec at 95°C and 60 sec at 60°C. Each qPCR run included negative control reactions without template. The specificity of each PCR amplification was confirmed by melting point analysis. The real-time PCR curves were analyzed by using the ABI PRISM 7000 SDS software (version 1.1). The initial template copy number of each transcript was calculated from a transcript-specific calibration curve, which was generated by amplifying a series of seven ten-fold dilutions of the respective plasmid standard (see above).

### Statistical analysis

The SPSS software package, version 16.0 (SPSS, Munich, Germany) was used for statistical analyses. The statistical significance of differences between two embryo groups was assessed by the Mann-Whitney U-test.

## Results

### Recovery of bovine embryos *in vitro* and *in vivo*

In total, we performed 17 independent *in vitro* fertilization (IVF) experiments, at least three for each observation time point (Table 2). Thereby, a total of 4,606 *in vitro* matured (IVM) oocytes were fertilized. The cleavage rate evaluated on day 3 was 88%±5% (mean value ± SD), the blastocyst rate was 25%±7% at day 6 and 37±5% at day 7. At harvesting, all oocytes/embryos were analyzed under a stereomicroscope by the same experienced investigator and classified according to their developmental status. The data are presented in Table 3. Unfertilized and uncleaved oocytes and embryos classified as severely retarded, degenerated or non-viable were not further analyzed.

**Table 2:** *In vitro* production of bovine embryos.

Time in culture*	Experiments	COCs°	Cleavage rate (%)	Blastocyst rate (%)
72 h (3 days)	3	514	80±7	
96 h (4 days)	3	843	90±4	
120 h (5 days)	4	1,244	90±2	
144 h (6 days)	3	1,006	88±3	25±7
168 h (7 days)	4	999	90±1	37±5
all	17	4,606	88±5	32±6

*after addition of frozen-thawed sperm;

° number of potentially fertilized cumulus-oocyte-complexes (COCs) used for *in vitro* maturation (IVM), fertilization (IVF), and culture (IVC).

**Table 3:** Stereomicroscopic classification of bovine embryos produced *in vitro*.

Time point*	Stereomicroscopic classification	Number	*Percent*
Day 3 (72 h)	UFOs°	109	*21*
	2–7 cells	171	*33*
	8–12 cells	162	*32*
	>12 cells	72	*14*
	all	514	*100*
Day 4 (96 h)	UFOs°	83	*10*
	retarded/degenerated/non-viable	147	*17*
	6–12 cells	222	*26*
	13–20 cells	243	*29*
	>20 cells	148	*18*
	all	843	*100*
Day 5 (120 h)	UFOs°	132	*11*
	retarded/degenerated/non-viable	429	*34*
	6–20 cells	274	*22*
	pre-compacted morula	366	*29*
	compacted morula	47	*4*
	all	1,248	*100*
Day 6 (144 h)	UFOs°	119	*12*
	retarded/degenerated/non-viable	425	*42*
	compacted morula	209	*21*
	early blastocyst	166	*17*
	non-expanded blastocyst	87	*9*
	all	1,006	*100*
Day 7 (168 h)	UFOs°	89	*9*
	retarded/degenerated/non-viable	550	*55*
	non-expanded blastocyst	139	*14*
	expanded blastocyst	154	*15*
	hatching blastocyst	67	*7*
	all	999	*100*

*after addition of frozen-thawed sperm;

° UFOs: unfertilized or uncleaved oocytes.

To validate our *in vitro* observations regarding their *in vivo* relevance, we performed two independent experiments using the same set of ten donor heifers to produce bovine embryos *in vivo* by superovulation (SO) and artificial insemination (AI). A total of 218 oocytes and embryos were harvested by uterine flushing 6–7 days after AI. The data from the stereomicroscopic analysis and classification are presented in Table 4. 53 compacted morulae/early blastocysts (i.e. without a clearly defined ICM) and 78 pre-hatched blastocysts classified as suitable for embryo transfer were chosen for further analyses. The average number of embryos analyzed per donor and superstimulation cycle was 6.5±1.4.

**Table 4:** Stereomicroscopic classification of bovine embryos produced *in vivo*.

Time point*	Stereomicroscopic classification	Number	*Percent*
Day 6–7	UFOs°	56	*25*
(159 h/168 h/183 h)	retarded/degenerated/non-viable	31	*15*
	compacted morula/early blastocyst	53	*25*
	non-expanded/expanded blastocyst	78	*35*
	all	218	*100*

*after three artificial inseminations within 24 hours;

° UFOs: unfertilized/uncleaved oocytes.

### Cell numbers and occurrence of cell death in bovine embryos produced *in vitro*

**Figure 2:**
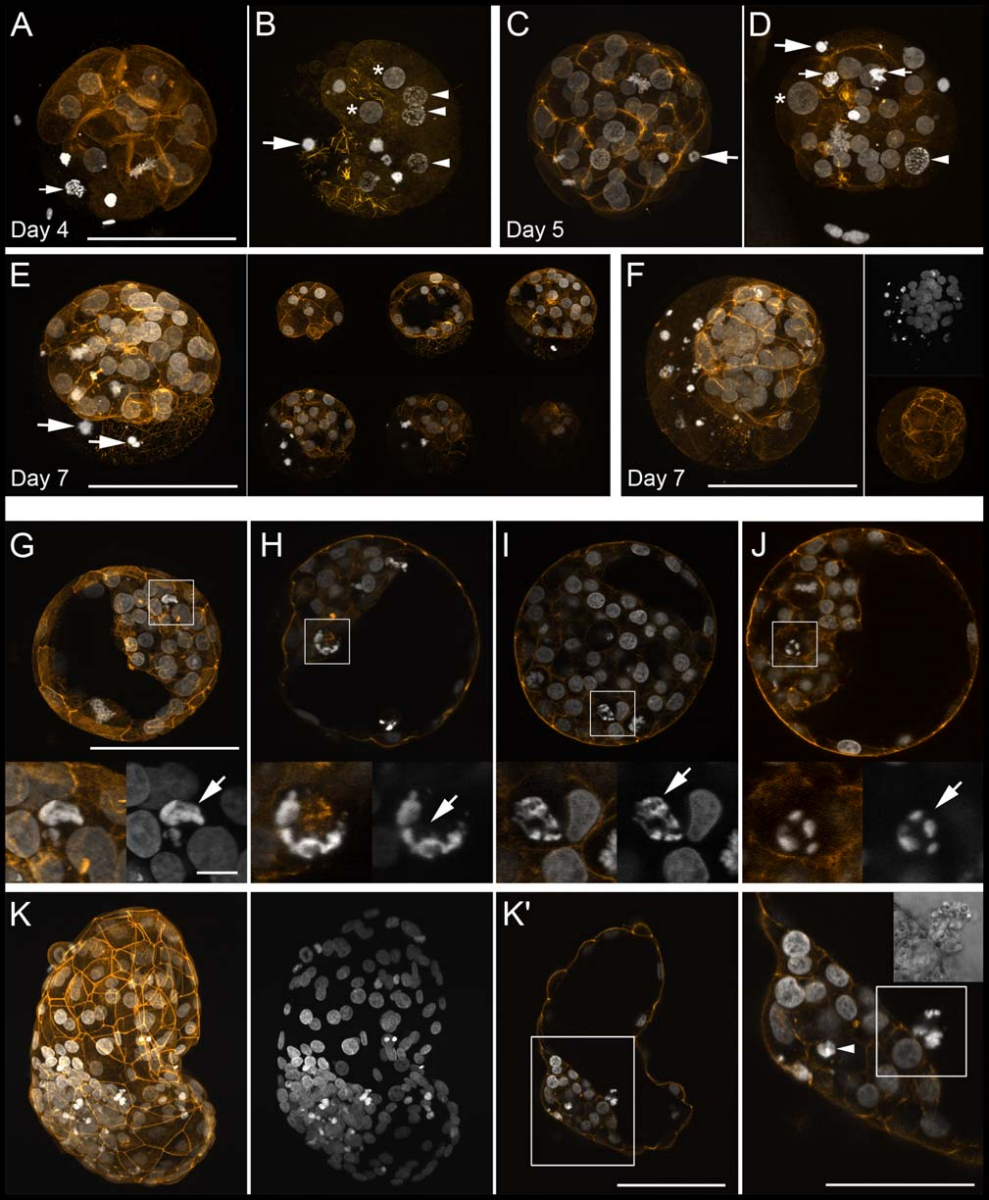
CLSM analysis of bovine IVF embryos. The embryos were fixed and mounted on coverslips in such a way that the three-dimensional structure was maintained. DNA staining with DAPI is shown in white, f-actin filaments (phalloidin-TRITC) in orange. Scale bars represent 100 µm (overviews) or 10 µm (details). **A–F:** Arrest and death of early blastomeres during the first four cleavage cycles. **A–D:** Maximum intensity z-projections of optical serial sections of embryos examined at day 4 (**A**, **B**) or day 5 (**C**, **D**). Many embryos show large early blastomeres that are arrested at interphase (asterisks) or prophase (arrow heads) or already show clear signs of cell death: DAPI staining reveals variably sized and irregularly shaped clumps of highly condensed chromatin (large arrows). Notably, frequent findings are remnants of mitotic chromosome structures (small arrows). **E**, **F:** Two day 7 embryos that initially survived the death of large early blastomeres. Remnants of early blastomere death can be seen until blastocyst hatching. Panel **E** shows an embryo in the form of a single maximum intensity z-projection of the entire confocal image stack (left) and as a sequence of six projections of 20 µm image sub-stacks (right). Panel **F** presents a z-projection (overlay image and separate channel images) through an entire embryo. **G–L:** Cell death in the inner cell mass of bovine IVF blastocysts examined at day 7: In expanding and hatching blastocysts, DAPI staining reveals highly condensed chromatin structures that are irregularly shaped and variable in size referring to different modes and stages of cell death (arrows). **G–J:** Four expanding blastocysts. **G** is a maximum intensity z-projection of a 20 µm image stack, **H**, **I**, **J** are single optical sections. Dying/dead cells are also shown at higher magnification. **K**, **K′:** Hatched blastocyst. Panel **K** presents a maximum intensity z-projection of a stack spanning the entire embryo (overlay image and DAPI alone). **K′:** Single optical section of the same blastocyst and enlarged view of the inner cell mass: Note a dying/dead cell (arrow head) in the interior of the inner cell mass as well as the extrusion of a dead cell into the blastocoel (insert: transmission light image).

The cell numbers and the incidence of cell death were determined by analyzing three-channel image stacks of whole-mount embryos (Figure 2). Serial transmission light images provided important structural information on the embryo architecture, on the size and shape of the embryonic cells, intracellular vacuoles, cytoplasmic borders and intercellular cavities. A series of embryos was stained with phalloidin-TRITC to visualize the f-actin cytoskeleton. The cell numbers were assessed by counting DAPI-stained cell nuclei and mitotic figures. Condensed and fragmented cell nuclei as well as chromatin remnants of mitotic figures were defined and counted as dead. In addition, the TUNEL assay was used to visualize DNA fragmentation, a frequently used parameter of apoptotic cell death, *in situ*. The TUNEL images were analyzed in comparison with the DAPI images. The result of the analysis of more than 25,000 nuclei is shown in Table S1. In total, only 33% of the cells with unequivocal morphological signs of cell death in the DAPI image were TUNEL positive. Notably, highly condensed as well as fragmented nuclei were completely negative, weakly or intensely stained. Moreover, we observed morphologically intact cell nuclei and grossly normal mitotic figures with weak to moderate TUNEL staining. As a consequence, we determined the proportion of dead cells only based on the morphological analysis of the DAPI images.

**Figure 3:**
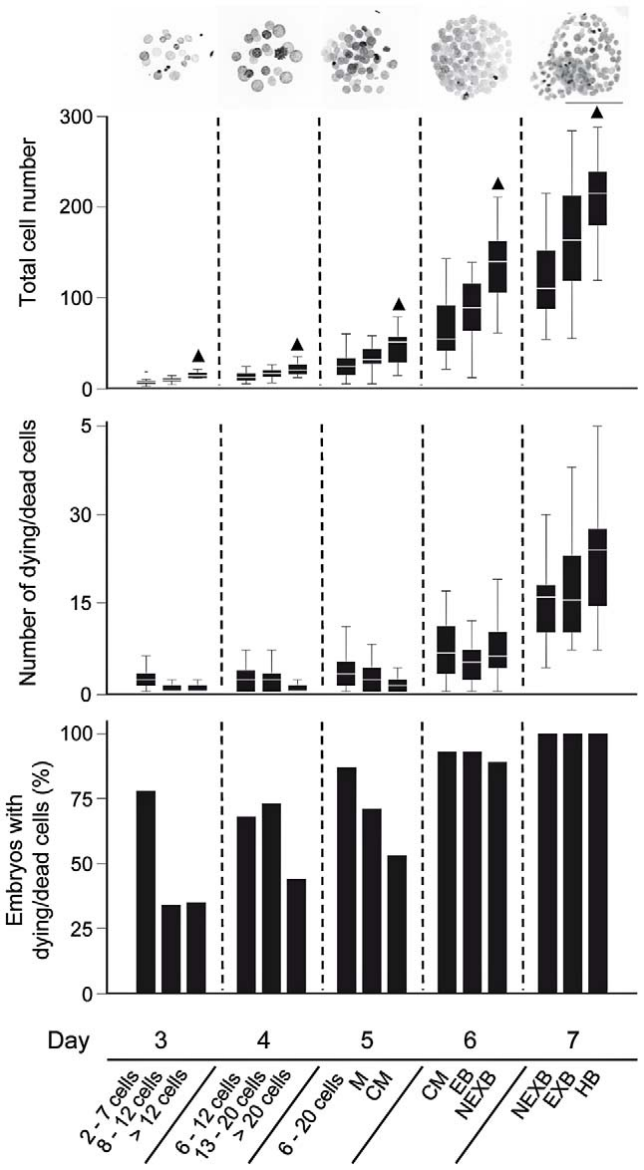
Cell numbers and occurrence of cell death in bovine embryos *in vitro*. Embryos were harvested 3 (72 h), 4 (96 h), 5 (120 h), 6 (144 h) and 7 (168 h) days after the addition of the sperm. Initially, the embryos were classified according to their developmental stage under a stereomicroscope. DAPI staining and CLSM were used to assess the number of intact and dying/dead cells (upper two diagrams) in three-dimensionally preserved specimens (see also Figure 2). The box plots show median values (white bars), 25th and 75th percentiles (boxes), 5th and 95th percentiles (whiskers). The data for each developmental stage were derived from three independent experiments. Note the high proportion of embryos with at least one dying/dead cell (lower diagram). The most advanced embryos at each time point (indicated by ▴) were used to obtain “normal” age- and stage-specific transcript copy number profiles *in vitro* (presented in Figure 5 and Figures S1 and S2). M = morula; CM = compacted morula; EB = early blastocyst; NEXB = non-expanded blastocyst; EXB = expanded blastocyst; HB = hatching/hatched blastocyst; Scale bar = 100 µm.

Embryo cell numbers and the incidence of dead cells observed at the different time points and developmental stages are shown in Figure 3 and Tables S2 and S3.

At day 3, the cell number per embryo varied between 2 and 21, with a median of 10. Notably, 48% of all day 3 embryos analyzed and 35% in the developmentally most advanced group of embryos already contained at least one dead cell (Figure 3). At day 4, the cell number ranged between 5 and 35 with a median of 16. At day 5, after 120 h, the fastest embryos had reached the compacted morula stage. Their cell number ranged between 14 and 79 with a median of 51. The most developed embryos harvested at day 6, after 144 hours, were non-expanded blastocysts with a fully formed blastocoel cavity and a clearly visible inner cell mass. The percentage of embryos with at least one dead cell increased from 48% on day 3 to 92% on day 6. On day 7 (168 h after addition of sperm) only blastocysts with a clearly visible ICM and blastocoel were included in the analysis. Notably, all day 7 blastocysts analyzed contained dead cells. Moreover, we consistently found noticeable proportions of dying/dead cells in the ICM of the most advanced embryos, namely hatching blastocysts with a well-developed and morphologically intact trophoblast (TB). Notably, CLSM analysis disclosed morphological features of different modes of ICM cell death in non-expanded, expanded and hatching/hatched blastocysts: diversely shaped structures of densely compacted chromatin which were in part compatible with apoptosis and in part clearly referred to mitotic cell death (Figure 2C and D).

**Figure 4:**
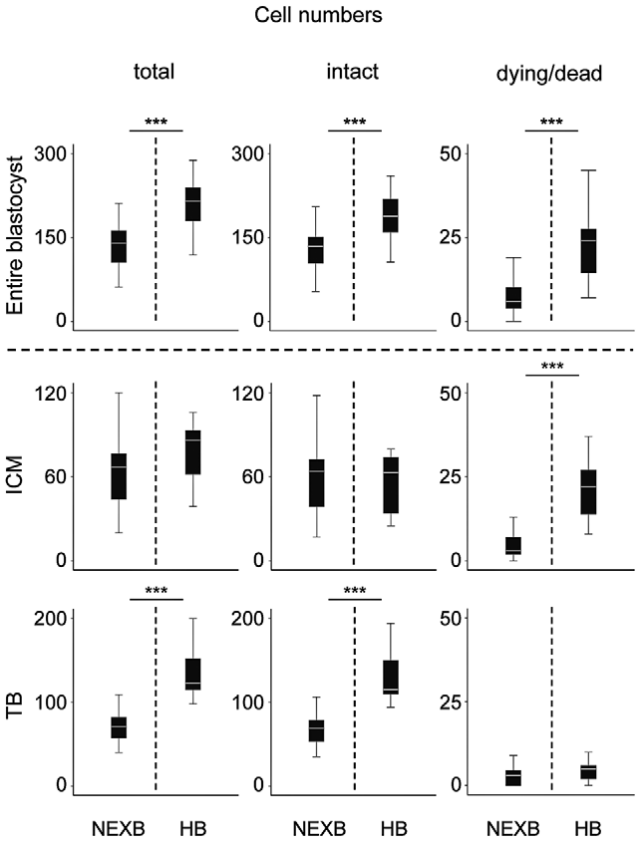
Cell numbers and occurrence of cell death in blastocysts produced *in vitro*. Embryos reaching the non-expanded blastocyst stage at day 6 and the stage of the hatching/hatched blastocyst at day 7, most likely represent undisturbed and “normal” development. The box plots show median values (white bars), 25th and 75th percentiles (boxes), 5th and 95th percentiles (whiskers). The data were obtained from three independent experiments. The increase of the total cell number was primarily due to the growth of the trophoblast. Note the striking increase of dying/dead cells in the ICM, while the median number of intact ICM cells remained constant at 64. Significant differences are marked by *** (p<0.001) as assessed by the Mann-Whitney U-test. ICM = inner cell mass; TB = trophoblast; NEXB = non-expanded blastocysts (day 6); HB = hatching/hatched blastocysts (day 7).

Non-expanded blastocysts were observed both at day 6 and day 7 (144 and 168 hours after addition of sperm), expanded and hatching blastocysts only on day 7. The blastocysts of each of the three stereomicroscopically defined stages were highly heterogeneous with respect to (a) the total embryo cell number, (b) the absolute number and proportion of ICM cells and (c) the incidence of dying/dead cells. The data are shown in Figure 4 and Table S4. Compared to the non-expanded blastocysts observed on day 6, non-expanded blastocysts harvested on day 7 were characterized by a lower proportion of ICM cells (p<0.05) and a higher proportion of dying/dead cells in the ICM (p<0.001).

The most advanced embryos at each time point most likely represent undisturbed and normal development. Therefore, the comparison of non-expanded day 6 blastocysts versus hatching day 7 blastocysts should provide valuable information on the physiological incidence and potential role of programmed cell death during normal blastocyst formation. The total cell number of non-expanded day 6 blastocysts ranged between 59 and 197 (median 140), while hatching day 7 blastocysts contained between 119 and 288 cells (median 217; Table S4). The increase in cell number from non-expanded to hatching blastocysts was primarily due to the growth of the TB (Figure 4). The median number of ICM cells in non-expanded day 6 blastocysts was 67 (range 20–130) and increased to 86 (range 39–106) in hatching day 7 blastocysts. The median proportion of ICM cells in non-expanded day 6 blastocysts was 47% (range 26–62%), and decreased to 39% (range 16–46%) in hatching day 7 blastocysts.

In the ICM of hatching day 7 blastocysts we found a significantly (p<0.001) higher proportion of dying/dead cells (median 27%, range 12–60%) compared to only 6% (range 0–35%) in non-expanded day 6 blastocysts (Table S4). Thereby, the median number of intact ICM cells (total ICM cell number minus the number of dying/dead ICM cells) remained constant at 64 (Figure 4). Notably, the proportion of dying/dead cells in the TB and the subzonal space was consistently very low with median values around 5%.

### Occurrence of cell death in embryos derived *in vivo*

To validate the relevance of our *in vitro* findings for the *in vivo* situation, we compared IVF embryos harvested on day 6 and 7 with embryos derived *in vivo* from superovulated and artificially inseminated donor animals (see experimental procedures). To obtain an adequate sample size for the *in vivo* embryos we merged the data from non-expanded and expanded blastocysts. Notably, the blastocysts produced *in vivo* revealed heterogeneity with respect to cell number and proportion of dead cells, although the variation appeared to be smaller than in their *in vitro* counterparts. The total cell number of non-hatched blastocysts ranged between 38 and 284 (median 143) *in vitro* (n = 85) and between 87 and 180 (median 133) *in vivo* (n = 19). The corresponding numbers of ICM cells were 15 to 139 (median 61) for *in vitro* and 30 to 81 (median 59) for *in vivo* produced embryos. Both *in vitro* and *in vivo* blastocysts contained considerable numbers of dying/dead cells in the ICM, with median proportions of 16% and 17%, respectively. The median incidence of dying/dead cells in the TB and/or at the embryo surface was only 5% *in vitro* and 3% *in vivo*.

### Cell division arrest und death of early blastomeres

After fertilization, the zygote is divided by a series of mitotic cleavage divisions into smaller and smaller cells - the blastomeres. In mammals, the daughter cells of each cleavage division are practically equal in size. Accordingly, the size of a blastomere is a function of the number of cell divisions passed and the first blastomere generations can be easily distinguished by their size. Developmentally delayed embryos typically/predominantly were composed of differently sized cells i.e. cells representing different cleavage cycles and blastomere generations. A high proportion of *in vitro* embryos harvested on days 3 to 6 contained at least one apparently arrested and/or dying/dead large cell of the first four blastomere generations (Figure 2A–F).

### Transcript copy numbers of apoptosis-related genes in bovine oocytes and embryos

**Figure 5:**
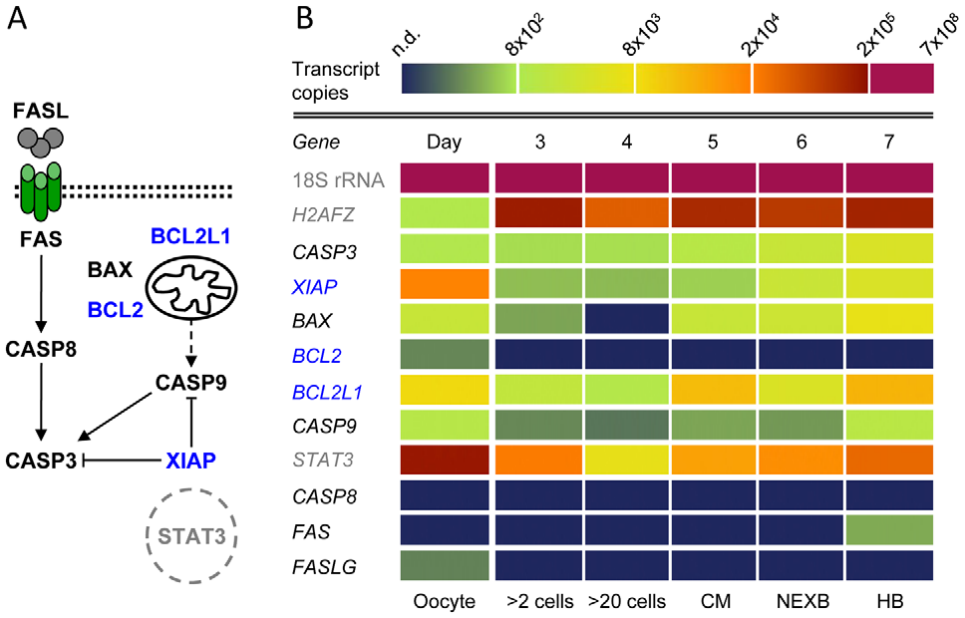
Transcript copy number profiles of apoptosis-related genes from the oocyte to the hatching blastocyst *in vitro*. **A:** The proteins encoded by the genes selected for analysis due to their decisive roles in the intrinsic or extrinsic initiation, in the regulation or execution of apoptosis. Pro-apoptotic proteins are shown in black, anti-apoptotic proteins are shown in blue. STAT3 seems to play a complex ambiguous role. **B:** Heatmap representation of transcript copy numbers per oocyte/embryo. Dark blue indicates values below the measurement limit (n. d. = not detected). Shown are the data (means of three independent experiments) from the most advanced embryos at each time point, which most likely represent “normal” development. 18S rRNA and H2AZF mRNA served as reference transcripts and are shown in grey. MC = compacted morulae; NEXB = non-expanded blastocysts; HB = hatching/hatched blastocysts.

We determined copy numbers of 18S rRNA and histone *H2AFZ* mRNA as well as transcript numbers of a set of ten apoptosis-related genes in bovine oocytes matured *in vitro* and embryos produced *in vitro* and *in vivo*. The genes selected for the analysis have been shown to play decisive roles in the initiation, regulation and execution of apoptosis (Figure 5A). RT-qPCR analysis was performed on total RNA extracted from pools of 10 *in vitro* matured oocytes, 10 *in vitro* produced embryos, or five *in vivo* derived embryos. The analysis included three (*in vitro* oocytes and embryos) or two (*in vivo* embryos) biological replicates, each from an independent experiment. Further details of the RT-qPCR analysis are presented in Table S5.

The most advanced embryos at each time point *in vitro* most likely represent an undisturbed and “normal” development and provide “normal” age- and stage-specific transcript copy number profiles *in vitro*. For simplification, in the following we restrict the description to the putatively “normal” transcript profiles. A synopsis of the transcript copy number profiles obtained for oocytes and fast developing embryos from day 3 until day 7 *in vitro* is given in Figure 5B: The total copy numbers per oocyte or embryo are visualized by a color-coded heat map. For each transcript, RT-qPCR analysis revealed a distinct abundance profile over time. Thereby, the abundance levels varied from gene to gene by several orders of magnitude. The results are presented in detail in Figures S1 (reference transcripts) and S2 (apoptosis-related genes).

The transcript copy number profiles for 18S rRNA (Figure S1), CASP3, XIAP, BAX, BCL2L1 (BCL-XL), CASP9, and STAT3 showed a decline from the oocyte until day 3 or day 4 to subsequently increase again (Figure S2). The number of mRNA copies for the histone H2AFZ showed a noticeable peak at day 3 prior to major genome activation followed by a transient decrease on day 4 (Figure S1). Transcripts for BCL2 were only in the oocyte unequivocally detected. Transcripts for the so-called extrinsic initiator caspase-8 were neither detected in oocytes nor in embryos up to the hatching blastocyst stage. Notably, transcripts for FAS-receptor (FAS) were only in hatching blastocysts clearly detected, transcripts for FAS-ligand (FASLG) only in the oocyte.

### Transcript copy numbers of blastocysts produced *in vivo*

To evaluate our findings in IVF embryos, the study was extended to *in vivo* blastocysts generated as described above. At the time point chosen for uterine flushing, no hatching or hatched blastocysts were recovered. To account for differences in the cell number we compared the transcript copy numbers per intact cell obtained by dividing the mean copy number per embryo by the mean number of intact cells (Table 5). The copy numbers per intact cell of 18S rRNA and *H2AFZ* mRNA were higher in the *in vivo* derived blastocysts than in their *in vitro* counterparts, however statistical significance was only reached in the case of *H2AFZ* (p<0.05). Further, in the *in vivo* blastocysts the transcript levels for XIAP (p<0.05), BAX, BCL2L1, and CASP9 (all p<0.01) were higher, whereas mRNA levels for CASP3 were higher in the *in vitro* embryos (p<0.05). Transcripts for BCL2, CASP8 and FASLG were not detected in both groups of embryos, *FAS* transcripts were detected only in the *in vivo* group at very low abundance. In general the transcript levels of apoptosis-related genes were very low in both groups of blastocysts investigated.

**Table 5:** Transcript copy numbers in non-expanded/expanded blastocysts *in vitro* and *in vivo*.

Transcript	Copy number per intact cell°				*p*
	*In vitro*		*In vivo*		
18S rRNA	2.5×10^6^	(1.4×10^6^)	8.0×10^6^	(3.7×10^6^)	
*H2AFZ*	570	(220)	1,400	(620)	0.05
*CASP3*	23	(12)	11	(4)	0.05
*XIAP*	30	(10)	46	(13)	0.05
*BAX*	30	(20)	100	(40)	0.01
*BCL2*	*n.d.*		*n.d.*		
*BCL2L1*	36	(20)	130	(40)	0.01
*CASP9*	4	(1)	13	(5)	0.01
*STAT3*	135	(50)	140	(80)	
*CASP8*	*n.d.*		*n.d.*		
*FAS*	*n.d.*		2	(3)	
*FASLG*	*n.d.*		*n.d.*		

The table shows means and standard deviations (in brackets) from 3 (*in vitro*) or 2 (*in vivo*) biological replicates.

° The copy number per intact cell was calculated by dividing the mean copy number per embryo by the mean number of intact cells (n = 128 for *in vitro* and n = 117 for *in vivo* embryos). Significance of differences was tested using the Mann-Whitney U test.

*n. d.* = not detected or transcript copy numbers below the measurement limit.

## Discussion

Our study of early bovine embryogenesis from fertilization to the hatching blastocyst addressed the occurrence and nature of cell death by 3D confocal laser scanning microscopy (3D CLSM) and used RT-qPCR to screen for the presence of transcripts for genes involved in classical cell death pathways.

### Developmental heterogeneity under standardized conditions *in vitro*

The developmental heterogeneity of *in vitro* produced embryos grown under identical conditions appears to reflect the variable developmental competence of individual oocytes, although an influence of stochastic events cannot be excluded. The latter assumption is supported by the observation of highly stochastic gene expression profiles in individual blastomeres of preimplantation mouse embryos [18].

Even when performed by experienced investigators, morphological assessment of individual embryos by standard bright field stereomicroscopy is rather imprecise and only very roughly reflects the actual developmental status and cellular composition. Cell nuclei and mitotic figures cannot be analyzed. In case of cytoplasmic fragmentation or cytokinesis failures and the formation of bi- and multinucleated cells, stereomicroscope-based cell number estimates can differ considerably from CLSM-based counts of cell nuclei and mitotic figures (see Table S2). We performed for the first time a comprehensive 3D CLSM study of a large number of bovine embryos *in toto*. This analysis revealed variably and irregularly shaped structures of densely compacted chromatin representing different stages of various types of cell demise (Figure 2). The occurrence of different forms of cell death variably encompassing different forms and degrees of DNA fragmentation is the most parsimonious explanation that only a subset of cell death is identified by the TUNEL assay.

### Cell cycle arrest and death of early blastomeres

The fate of the embryo depends on the outcome of the first four cleavage divisions that have a high failure rate. A considerable proportion of fertilized oocytes fail as a consequence of errors during this early phase of development. These can lead to immediate embryo death, the arrest of single blastomeres or to aberrant subsequent development. Frequent findings are different types of interphase arrest, different forms of mitotic arrest in pro-, meta-, ana- and telophase as well as mitotic cell death (Figure 2A–D). Often, the first three and four cleavage divisions result in less than eight and sixteen viable cells respectively that continue to divide. The different types of cell cycle arrest during the first cleavage cycles are variably linked to various forms of cell degradation. In this study, nearly 50 percent of the *in vitro* embryos analyzed on day 3 already contained at least one cell with morphological signs of advanced cell death (see above). Importantly, the actual incidence of early blastomere loss may even be underestimated: early blastomeres that are permanently arrested in interphase or mitosis can remain morphologically intact and seemingly viable until day 7. Irreversibly arrested as well as dying and dead early blastomeres remain outside/separate from the developing embryo in the subzonal space and are not engulfed by the developing embryo. Both arrested early blastomeres and the remnants of early blastomere death are to be seen up to the hatching blastocyst stage (Figure 2E and F).

Many embryos that initially survive have to compensate the loss of one or more early blastomeres and their progeny. Moreover, irregular mitotic figures according to centrosome and mitotic spindle aberrations, mitotic cell death and signs of nuclear atypia refer to mosaic aneuploidy and chromosomal instability. The decision whether the loss of early blastomeres can be compensated may depend on the epigenetic commitment of the surviving cells that continue to divide. At present, it is not clear, when the process of epigenetic diversification starts in bovine embryos. As an example, we don't know whether the four blastomeres generated by the second cleavage cycle or the eight cells generated by the third cleavage cycle are epigenetically equivalent or are already restricted in their developmental potential. An important question is whether early blastomere loss has long-term effects on the further development even when the surviving cells have a normal genotype/epigenotype.

### Developmental programmed cell death in the ICM of regularly developing hatching blastocysts

*In vitro* blastocysts of the same age had widely varying cell numbers of both ICM and TB. The incidence of cell death in the TB was consistently very low, but highly variable in the ICM. *In vitro* development of the non-expanded (day 6) to the hatching blastocyst (day 7) was characterized by a rapid increase in the TB cell number. The number of intact ICM cells remained rather constant, while the proportion of dying/dead ICM cells increased. Remarkably, the highest incidence of ICM cell death was observed in seemingly well developed hatching blastocysts. Thus, the development from the non-expanded to the hatching blastocyst seems to coincide with the onset of a physiological wave of controlled ICM cell death due to a temporary limitation of the ICM cell number and/or developmental cell selection/sorting processes. Intriguingly, there is rather vague evidence that ICM cell death is mainly due to classical caspase-mediated apoptosis. There are no quantitative microscopic studies that document massive activation of effector caspases in the majority of dying/dead ICM cells. Our CLSM analysis disclosed morphological features of different modes of cell death: diversely shaped structures of densely compacted chromatin that in part are compatible with apoptosis and in part clearly refer to mitotic cell death (Figure 2G–K). Concordant with the coincident occurrence of different cell death modes, the TUNEL assay revealed DNA fragmentation only in a subset of ICM cells with advanced features of cell death. Transmission electron microscopy was not helpful to unravel the enigma of ICM cell death: As others [9], [19], we detected some morphological features typical of apoptosis, such as nuclear condensation and fragmentation, as well as structures that can be interpreted as engulfment of cell corpses by neighboring cells (data not shown). A quantitative evaluation of cell death by TEM is practically impossible. Moreover, since only a limited number of embryos and a small number of cells can be analyzed by TEM, there is a serious risk of false interpretation or over-interpretation of artifactual, rare and non-representative findings. Morphological evidence for different types of cell death is not necessarily contradictory to the occurrence of developmentally programmed cell death: Recent research has conclusively shown that there is a broad spectrum of different cell death subroutines that causes a serious nomenclature problem. The textbook simplification ‘programmed cell death = apoptosis = caspase activation’ has become more and more questionable (for reviews see [20], [21]).

### Cell death in embryos derived *in vivo*

To what extent is ICM cell death indicative for altered development *in vitro*? Several studies of bovine blastocysts reported higher cell death indices *in vitro* than *in vivo* [8], [11], [22]. However, the published data on the incidence of cell death in bovine embryos are difficult to compare due to different detection methods and quantification criteria. In our study, CLSM analysis of blastocysts produced *in vivo* revealed practically the same degree of developmental heterogeneity regarding the cell numbers and the occurrence of cell death as observed in embryos produced *in vitro*. One has to consider that all embryos in a culture well *in vitro* have practically the same age that is quite exactly defined by the time of sperm addition. In contrast, embryos produced *in vivo* represent a broader time window due to asynchronous ovulation and fertilization. Failures of the first cleavage divisions leading to early blastomere arrest and death are a notably frequent finding also in *in vivo* embryos. Further, *in vivo* produced blastocysts showed practically the same high incidence of ICM cell death as their *in vitro* counterparts supporting the concept of developmentally programmed forms of ICM cell death. We have to admit that at present there is no empirical evidence that an apoptosis index might be a suitable parameter to predict the developmental potential of blastocysts. Future studies need to elucidate the modes and mechanisms of ICM death to understand the fundamental principles of how ICM cells start to build up a new organism.

### The analysis of transcript abundances in mammalian oocytes and early embryos

In parallel to the morphological analyses, we analyzed the mRNA abundance of selected apoptosis-associated genes in bovine oocytes and pre-implantation embryos by quantitative RT-PCR. The analysis of transcript levels is a first step to elucidate which components of the apoptosis machinery may be present and play a role in early embryo development. In small pools of oocytes and embryos, we simultaneously studied a set of genes, which are known be critically involved in the execution and regulation of apoptosis as well as in the induction of apoptosis by intrinsic and extrinsic signaling pathways. Instead of relative transcript abundances, we determined transcript copy numbers in relation to embryonic age and developmental stage. Moreover, we calculated transcript copy numbers per cell by dividing the copy numbers per embryo through the average cell numbers determined in parallel for the respective embryo age and developmental stage.

Mammalian oocytes contain large stockpiles of translationally inactive mRNAs with short poly(A) tails. After fertilization, such maternal mRNAs are activated for translation by cytoplasmic polyadenylation to drive the first cleavage divisions. Regulation of the poly(A) tail length appears to be the major mechanism for the regulation of mRNA translation after fertilization (for review see [23], [24]). In the following, the maternal transcripts are gradually degraded in parallel to the gradual activation of the embryonic genome and the increase in embryonic transcripts [23], [25]. Reverse transcription using random-hexamer (RH)-primers includes all maternal and embryonic transcripts irrespective of the presence of a poly(A) tail.

A major challenge of transcript analyses in mammalian oocytes and preimplantation embryos is the relatively low RNA content. Estimates for bovine oocytes and blastocysts based on Northern blot analyses are in the range of 1–2 ng and 5 ng, respectively [26]. Accordingly, particularly critical issues are the efficiency and reproducibility of RNA extraction and reverse transcription (for review see [27]). Since early embryogenesis from fertilization to the blastocyst stage is characterized by extremely dynamic cellular and molecular changes, the choice of appropriate reference genes for this developmental window is a problem difficult to solve (for discussion see [28], [29], [30]). Absolute quantification of transcript abundances avoids the problem of adequate reference genes for normalization.

The mere detection of transcripts and the observation of changes in relative transcript levels can only provide rather limited evidence for a functional relevance. Nevertheless, the determination of absolute transcript abundances is essential to define the normal range of specific transcript levels and to gain insights into the regulation of the expression of specific genes, e.g., aided by computer simulations.

### Transcript copy number profiles of apoptosis-related genes in bovine oocytes and early embryos derived *in vitro*

This study provides for the first time copy numbers for 18S rRNA and *H2AFZ* mRNA during early bovine development from the oocyte to the hatching blastocyst. rRNA synthesis has been shown to start during the third cleavage cycle of bovine development [31], while the ultrastructure and molecular composition of functional nucleoli has been reported to be established during the fourth cell cycle [32]. Accordingly, we found a steep increase in 18S rRNA on day 5 at the morula stage. H2AFZ is a highly conserved variant of histone H2A. H2AFZ seems to play a complex role in epigenetic/chromatin-based gene regulation, but its precise function is not yet well defined [33]. In matured oocytes, the transcript copy number for H2AFZ was very low. Notably, there was an explosive peak of the copy number per embryo on day 3 after fertilization – preceding major genome activation.

CASP3 is the major effector caspase of the apoptosis system (for review see [34]) and appears to have multiple functions in cell survival, proliferation, differentiation and inflammation (for review see [35]). Interestingly, a critical role for CASP3 in embryonic stem cell differentiation has been proposed [36]. In mice, homozygous knockout of *Casp3* results in fetal and perinatal death (for reviews see [37], [38]), however the role of CASP3 in early development is not yet clear. Studies in cattle and other mammalian species found *CASP3* transcripts in oocytes and embryos up to the hatching blastocyst [39], [40], [41]. In our study, we also consistently found *CASP3* transcripts from the oocyte to the blastocyst stage, but at very low levels. This raises the question, whether a sufficient number of pro-caspase-3 molecules required for the execution of apoptosis may be present in early bovine embryos.

XIAP (BIRC4) protein is a direct inhibitor of caspases 3, 7 and 9 and a potent suppressor of apoptosis (for review see [42]). Notably, we found high *BIRC4* transcript copy numbers in oocytes, while only one tenth of the oocyte transcript level was detected in day 3 embryos. During further development to the hatching blastocyst, the transcript copy numbers per embryo for XIAP and CASP3 were in the same range. Notably, important not apoptosis-related functions have been proposed for BIRC4 and the other members of the IAP family e.g. in cell cycle regulation, protein degradation and caspase-independent signal transduction cascades (for review see [43]).

BAX protein is regarded as a key initiator of apoptosis by triggering the release of cytochrome c from mitochondria (for review see [44], [45]). For BAX and BCL2L1, an anti-apoptotic member of the BCL2 protein family, transcripts were consistently found in both oocytes and all embryonic stages analyzed. Thereby, the transcript copy numbers for both genes were in the same range. In contrast, transcripts for the (anti-apoptotic) protein BCL2 were only in matured oocytes at very low levels consistently detected.

Similar as for *CASP3* mRNA (see above), very low transcript copy numbers were found for CASP9, the central initiator caspase of the intrinsic apoptosis induction pathway and activator of CASP3 (for reviews see [37], [38]). In non-expanded day 6 blastocysts only few copies per cell were measured, casting doubts on intrinsic activation of caspase-mediated cell death.

The absence or very low transcript copy number (under the measurement limit of 200 copies per oocyte or embryo) for FAS-receptor and FAS-ligand before blastocyst formation argue against a relevant role of the FAS/FASLG-extrinsic apoptosis induction pathway at this early stage of development.

Transcripts for pro-caspase-8 were neither detected in oocytes nor in embryos up to the hatching blastocyst stage. Since caspase-8 is involved in all known death receptor pathways (for review see [46]), it is unlikely that other death receptor signaling pathways induce apoptosis in early bovine embryos.

### Transcript abundances in blastocysts derived *in vivo*

As a first step to evaluate our findings on transcript copy numbers *in vitro*, we analyzed *in vivo* produced embryos in the same way (see above). To take into account differences in the cell number, we compared transcript copy numbers per cell additionally normalized against the two reference genes. Overall, the transcript copy numbers determined in the *in vivo* embryos were practically in the same range as those measured in the *in vitro* counterparts. Numerous studies have addressed differences in (relative) transcript abundances between *in vitro* and *in vivo* produced bovine embryos and found statistically significant differences for a broad range of genes (for reviews see [47], [48]). The interpretation of such differences in transcript abundances has to be very cautious. The comparison of gene expression pattern between *in vitro* and *in vivo* development is a difficult task: a major problem is to compare embryos of the same age and developmental stage (see above). Moreover, even highly statistical differences in transcript abundances not necessarily coincide with functionally relevant differences at the protein level. Finally, differences in transcript abundances between embryos grown under different conditions may simply reflect successful adaptation to different environmental conditions without long term effects for development.

### Conclusions and perspectives

After fertilization, the formation of a regular diploid genome and the maintenance of the genome integrity over the first cleavage divisions from the chromosome level down to the nucleotide level appear to be the primary critical task in mammalian embryogenesis [49]. In cattle, the first and the next three cleavage divisions have a high failure rate and are the main source of developmental heterogeneity *in vitro* and *in vivo*. Cell division errors at this early phase frequently cause immediate embryo death, irreversible cell cycle arrest and death of single blastomeres and/or aberrant subsequent development. During the development of the non-expanded to the hatching blastocyst, there seems to be a physiological wave of controlled ICM cell death keeping the ICM cell number constant for a while. Importantly, the dying/dead cells in the ICM only in part show morphological features compatible with apoptosis. The morphological findings as well as the absence or very low abundance of transcripts for CASP3, CASP9, CASP8 and FAS/FASLG are in conflict with the view that classical caspase-mediated apoptosis is the major cause of cell death in early bovine development.

Future studies need to disclose in detail the main failures of the first four cleavage cycles: the mechanisms underlying centrosome and spindle aberrations, the activity of cell surveillance/DNA repair systems and the role of cell cycle checkpoints. The investigation of the most frequent aberrations of the first cleavage cycles can guide us to understand the functional, structural and molecular basis of the most relevant oocyte deficiencies. At the blastocyst stage, the nature and causes of ICM cell death as well as the prognostic significance of the ICM cell number and ICM cell death need to be addressed.

## Supporting Information

Figure S1 **Copy number profiles of 18S rRNA and H2AFZ mRNA from the oocyte to the hatching blastocyst** ***in vitro*** **.** Shown are the data (means and standard deviations of three independent experiments) from *in vitro* matured oocytes and the most advanced embryos at each time point which most likely represent “normal” development *in vitro*. Transcript copy numbers per oocyte/embryo are presented on the left, and the corresponding values per cell on the right. Note that the y-axis scales vary.
(PDF)

Figure S2 **Transcript copy number profiles of apoptosis-related genes from the oocyte to the hatching blastocyst** ***in vitro*** **.** Shown are the data (means and standard deviations of three independent experiments) from *in vitro* matured oocytes and the most advanced embryos at each time point which most likely represent “normal” development *in vitro*. Transcript copy numbers per oocyte/embryo are presented on the left, and the corresponding values per cell on the right. Note the different scaling of the y-axis. CM = compacted morula; NEXB = non-expanded blastocyst; HB = hatching blastocyst.
(PDF)

Table S1 Quantitative analysis of cell death in bovine embryos produced *in vitro*: comparison of DAPI staining and TUNEL.
(PDF)

Table S2 Embryo cell numbers and the incidence of dying/dead cells *in vitro*.
(PDF)

Table S3 Proportion of *in vitro* produced embryos with dying/dead cells.
(PDF)

Table S4 Cell numbers and the incidence of dying/dead cells in blastocysts produced *in vitro*.
(PDF)

Table S5 Calibration curves/CT-values for quantitative Real-Time-PCR.
(PDF)
