# Supplementary material for: Cell Arrest and Cell Death in Mammalian Preimplantation Development: Lessons from the Bovine Model
Source: PLoS One. 2011 Jul 21;6(7):e22121. doi: 10.1371/journal.pone.0022121 (PMC3141016; doi:10.1371/journal.pone.0022121)
Supplement: Figure S1 — Copy number profiles of 18S rRNA and H2AFZ mRNA from the oocyte to the hatching blastocyst in vitro . Shown are the data (means and standard deviations of three independent experiments) from in vitro matured oocytes and the most advanced embryos at each time point which most likely represent “normal” development in vitro. Transcript copy numbers per oocyte/embryo are presented on the left, and the corresponding values per cell on the right. Note that the y-axis scales vary. (PDF) [file pone.0022121.s001.pdf]

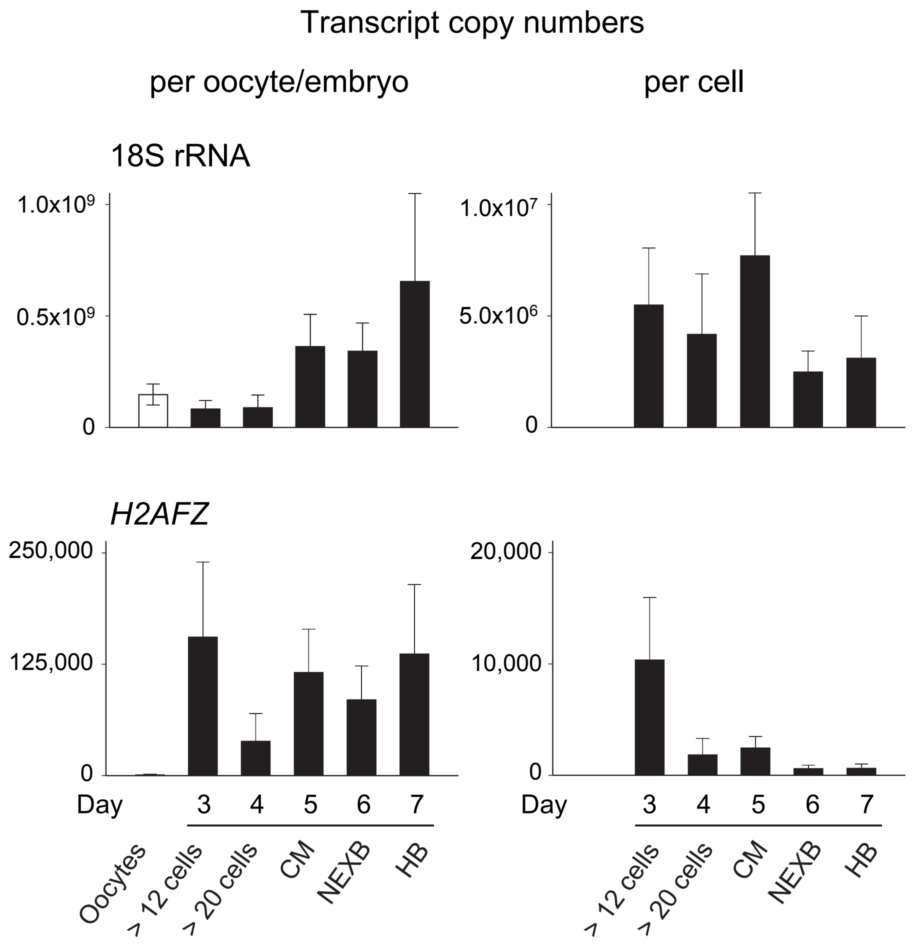

Figure S1. Copy number profiles of 18S rRNA and H2AFZ mRNA from the oocyte to the hatching blastocyst *in vitro*. Shown are the data (means and standard deviations of three independent experiments) from *in vitro* matured oocytes and the most advanced embryos at each time point which most likely represent "normal" development *in vitro.* Transcript copy numbers per oocyte/embryo are presented on the left, and the corresponding values per cell on the right. Note that the y-axis scales vary.
