# Supplementary material for: Cell Arrest and Cell Death in Mammalian Preimplantation Development: Lessons from the Bovine Model
Source: PLoS One. 2011 Jul 21;6(7):e22121. doi: 10.1371/journal.pone.0022121 (PMC3141016; doi:10.1371/journal.pone.0022121)
Supplement: Figure S2 — Transcript copy number profiles of apoptosis-related genes from the oocyte to the hatching blastocyst in vitro . Shown are the data (means and standard deviations of three independent experiments) from in vitro matured oocytes and the most advanced embryos at each time point which most likely represent “normal” development in vitro. Transcript copy numbers per oocyte/embryo are presented on the left, and the corresponding values per cell on the right. Note the different scaling of the y-axis. CM = compacted morula; NEXB = non-expanded blastocyst; HB = hatching blastocyst. (PDF) [file pone.0022121.s002.pdf]

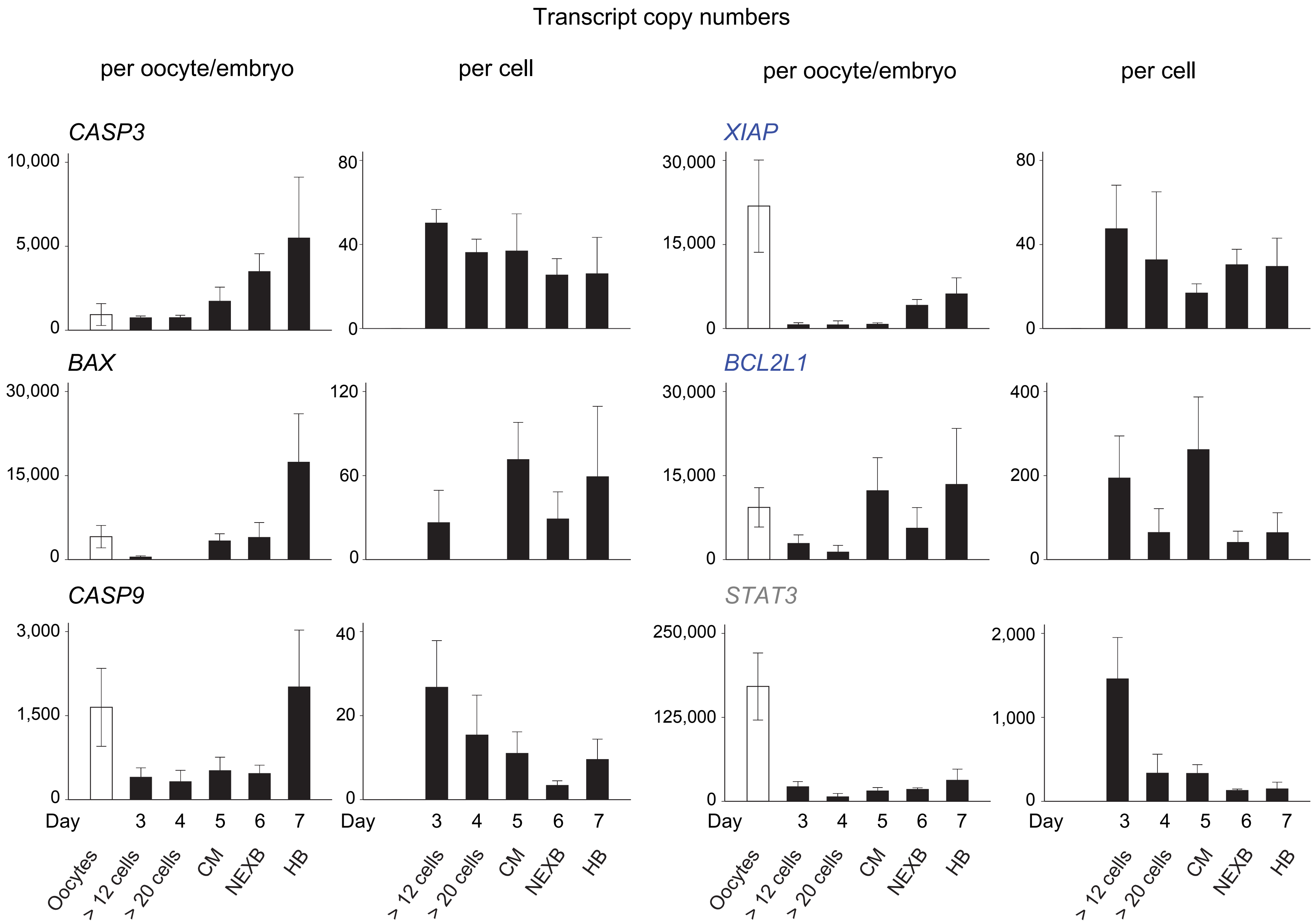

Transcript copy numbers
per oocyte/embryo
per cell
per oocyte/embryo
per cell
CASP3
XIAP
BAX
BCL2L1
CASP9
STAT3
10,000
5,000
0
80
40
0
30,000
15,000
0
80
40
0
30,000
15,000
0
120
60
0
30,000
15,000
0
400
200
0
3,000
1,500
0
40
20
0
250,000
125,000
0
2,000
1,000
0
Day
3
4
5
6
7
Oocytes
> 12 cells
> 20 cells
CM
NEXB
HB

Figure S2. Transcript copy number profiles of apoptosis-related genes from the oocyte to the hatching blastocyst *in vitro*. Shown are the data (means and standard deviations of three independent experiments) from *in vitro* matured oocytes and the most advanced embryos at each time point which most likely represent "normal" development *in vitro.* Transcript copy numbers per oocyte/embryo are presented on the left, and the corresponding values per cell on the right. Note the different scaling of the y-axis. CM = compacted morula; NEXB = non-expanded blastocyst; HB = hatching blastocyst.
