# Supplementary material for: Cell Arrest and Cell Death in Mammalian Preimplantation Development: Lessons from the Bovine Model
Source: PLoS One. 2011 Jul 21;6(7):e22121. doi: 10.1371/journal.pone.0022121 (PMC3141016; doi:10.1371/journal.pone.0022121)
Supplement: Table S1 — Quantitative analysis of cell death in bovine embryos produced in vitro: comparison of DAPI staining and TUNEL. (PDF) [file pone.0022121.s003.pdf]

Table S1. Quantitative analysis of cell death in bovine embryos produced *in vitro*: comparison of DAPI staining and TUNEL.

Time point*	Total number of cells	Number of dying/dead cells	
		DAPI (D+)	TUNEL (T+)°
Day 3 (72 h)	864	88	38 (43)
Day 4 (96 h)	1,378	147	46 (31)
Day 5 (120 h)	2,346	208	78 (38)
Day 6 (144 h)	8,197	567	216 (38)
Day 7 (160 h)	12,321	1,389	412 (30)
all	25,106	2,399	790 (33)

*after addition of frozen-thawed sperm; the cell numbers were assessed by counting DAPI-stained cell nuclei and mitotic figures: cells containing condensed, fragmented and degraded cell nuclei as well as chromatin remnants of mitotic figures were counted as dying/dead (D+); °number and percentage (in brackets) of cells with unequivocal morphological signs of cell death in the DAPI image, which were TUNEL-positive (T+).
