# Supplementary material for: Cell Arrest and Cell Death in Mammalian Preimplantation Development: Lessons from the Bovine Model
Source: PLoS One. 2011 Jul 21;6(7):e22121. doi: 10.1371/journal.pone.0022121 (PMC3141016; doi:10.1371/journal.pone.0022121)
Supplement: Table S2 — Embryo cell numbers and the incidence of dying/dead cells in vitro. (PDF) [file pone.0022121.s004.pdf]

Table S2. Embryo cell numbers and the incidence of dying/dead cells *in vitro*.

Time point* and stereomicroscopic classification	n°	Cell number per embryo		Percentage of dying/dead cells
		total	dying/dead	
**Day 3 (72 h)**				
2 - 7 cells	27	7; 7 ± 3; 2 - 18	2, 2 ± 2; 0 - 10	25; 33 ± 26; 0 - 88
8 - 12 cells	29	9; 9 ± 2; 4 - 16	0; 1 ± 1; 0 - 3	0; 5 ± 9; 0 - 33
> 12 cells	26	14; 15 ± 3; 11 - 21	0, 1 ± 1; 0 - 2	0; 3 ± 5; 0 - 17
all	82	10; 10 ± 4; 2 - 21	0; 1 ± 2; 0 - 10	0; 14 ± 21; 0 - 86
**Day 4 (96 h)**				
6 - 12 cells	28	13; 12 ± 5; 5 - 24	2; 2 ± 2; 0 - 7	17; 24 ± 29; 0 - 90
13 - 20 cells	30	17; 17 ± 5; 6 - 26	2; 2 ± 2; 0 - 7	12; 14 ± 16; 0 - 67
> 20 cells	25	20; 21 ± 6; 12 - 35	0; 1 ± 1; 0 - 5	0; 4 ± 7; 0 - 36
all	83	16; 16 ± 6; 5 - 35	1; 2 ± 2; 0 - 7	7; 15 ± 23; 0 - 90
**Day 5 (120 h)**				
6 - 20 cells	30	24; 27 ± 15; 5 - 66	3, 4 ± 4; 0 - 14	13; 21 ± 24; 0 - 85
pre-compacted morula	24	32; 35 ± 17; 5 - 77	2; 3 ± 4; 0 - 14	4; 15 ± 22; 0 - 73
compacted morula	15	51; 47 ± 19; 14 - 79	1; 2 ± 2; 0 - 9	2; 3 ± 4; 0 - 13
all	69	31; 34 ± 18; 5 - 79	2; 3 ± 2; 0 - 14	5; 15 ± 23; 0 - 85
**Day 6 (144 h)**				
compacted morula	30	60; 69 ± 34; 21 - 143	7; 7 ± 5; 0 - 17	9; 13 ± 14; 0 - 59
early blastocyst	29	89; 84 ± 32; 12 - 139	5; 5 ± 4; 0 - 15	7; 7 ± 6; 0 - 27
non-expanded blastocyst	27	140; 131 ± 40; 59 - 197	6; 8 ± 6; 0 - 24	5; 6 ± 5; 0 - 21
all	86	91; 94 ± 44; 12 - 197	6; 7 ± 5; 12 - 21	6; 9 ± 8; 0 - 59
**Day 7 (168 h)**				
non-expanded blastocyst	27	110; 120 ± 44; 54 - 215	16; 15 ± 6; 4 - 30	12; 13 ± 6; 5 - 27
expanded blastocyst	30	164; 169 ± 56; 49 - 284	16; 17 ± 8; 7 - 38	10; 11 ± 5; 3 - 25
hatching blastocyst	20	215; 218 ± 39; 143 - 288	24; 23 ± 10; 7 - 45	11; 11 ± 4; 4 - 18
all	77	159; 162 ± 60; 49 - 288	17; 18 ± 9; 4 - 45	11; 12 ± 5; 3 - 27

*after addition of frozen-thawed sperm;

°number of embryos analyzed; values are: median, mean ± standard deviation (SD); range.
