# Supplementary material for: Cell Arrest and Cell Death in Mammalian Preimplantation Development: Lessons from the Bovine Model
Source: PLoS One. 2011 Jul 21;6(7):e22121. doi: 10.1371/journal.pone.0022121 (PMC3141016; doi:10.1371/journal.pone.0022121)
Supplement: Table S3 — Proportion of in vitro produced embryos with dying/dead cells. (PDF) [file pone.0022121.s005.pdf]

Table S3. Proportion of *in vitro* produced embryos with dying/dead cells.

Time point* and stereomicroscopic classification	Number of embryos	Number (percentage) of embryos with at least one dying/dead cell
**Day 3 (72 h)**		
2 - 7 cells	27	21 (78)
8 - 12 cells	29	10 (34)
> 12 cells	26	9 (35)
all	82	40 (48)
**Day 4 (96 h)**		
6 - 12 cells	28	19 (68)
13 - 20 cells	30	22 (73)
> 20 cells	25	11 (44)
all	83	52 (62)
**Day 5 (120 h)**		
6 - 20 cells	30	26 (87)
pre-compacted morula	24	17 (71)
compacted morula	15	8 (53)
all	69	51 (74)
**Day 6 (144 h)**		
compacted morula	30	28 (93)
early blastocyst	29	27 (93)
non-expanded blastocyst	27	24 (89)
all	86	79 (92)
**Day 7 (168 h)**		
non-expanded blastocyst	27	27 (100)
expanded blastocyst	30	30 (100)
hatching blastocyst	20	20 (100)
all	77	77 (100)

*after addition of frozen-thawed sperm.
