# Supplementary material for: Cell Arrest and Cell Death in Mammalian Preimplantation Development: Lessons from the Bovine Model
Source: PLoS One. 2011 Jul 21;6(7):e22121. doi: 10.1371/journal.pone.0022121 (PMC3141016; doi:10.1371/journal.pone.0022121)
Supplement: Table S4 — Cell numbers and the incidence of dying/dead cells in blastocysts produced in vitro. (PDF) [file pone.0022121.s006.pdf]

Table S4. Cell numbers and the incidence of dying/dead cells in blastocysts produced *in vitro*.

**Time point* and stereomicroscopic classification**	**Number and *percentage*° of cells**			**Number and *percentage*° of dying/dead cells**		
	**total**	**ICM**	**TB**	**total**	**ICM**	**TB**
**Day 6 (144 h)**						
non-expanded blastocyst<br>(n = 27)	140; 131 ± 40^{a}; 59 - 197	67; 65 ± 27; 20 - 130<br>*47; 46 ± 9^{a}; 26 - 62*	71; 71 ± 20^{a}; 40 - 120<br>*53; 54 ± 9^{a}; 38 - 74*	6; 8 ± 6$^{a, b}$; 1 - 24<br>*5; 6 ± 5$^{a, b}$; 0 - 21*	3; 5 ± 4$^{a, b}$; 1 - 15<br>*6; 9 ± 9$^{a, b}$; 0 - 35*	3; 3 ± 3; 0 - 13<br>*4; 6 ± 6; 0 - 23*
**Day 7 (168 h)**						
non-expanded blastocyst<br>(n = 27)	110; 120 ± 44; 54 - 215	52; 51 ± 27; 22 - 100<br>*41; 41 ± 10; 19 - 59*	64; 70 ± 24; 34 - 117<br>*59; 59 ± 10; 41 - 81*	16; 15 ± 6^{b}; 4 - 30<br>*12; 12 ± 6^{b}; 5 - 27*	10; 10 ± 6^{b}; 1 - 24<br>*18; 21 ± 13^{b}; 1 - 67*	5; 5 ± 4; 0 - 13<br>*7; 8 ± 7; 0 - 27*
expanded blastocyst<br>(n = 30)	164; 169 ± 56; 49 - 284	66; 73 ± 31; 20 - 139<br>*42; 42 ± 8; 19 - 56*	92; 98 ± 30; 35 - 151<br>*58; 59 ± 8; 44 - 81*	16; 17 ± 8; 7 - 38<br>*10; 11 ± 5; 3 - 25*	12; 12 ± 6; 4 - 24<br>*20; 20 ± 10; 4 - 40*	4; 5 ± 3; 0 - 15<br>*4; 5 ± 4; 0 - 20*
hatching blastocyst<br>(n = 20)	217; 210 ± 44^{a}; 119 - 288	86; 77 ± 22; 39 - 106<br>*39; 36 ± 9^{a}; 16 - 46*	123; 140 ± 37^{a}; 98 - 228<br>*61; 64 ± 9^{a}; 54 - 84*	24; 25 ± 9^{a}; 12 - 45<br>*11; 12 ± 4^{a}; 4 - 18*	22; 21 ± 8^{a}; 8 - 37<br>*27; 28 ± 12^{a}; 12 - 60*	5; 4 ± 3; 0 - 10<br>*4; 3 ± 2; 0 - 8*

*after addition of frozen-thawed sperm; °lower row (in italics); values are: median; mean ± standard deviation (SD); range; significant differences ($p \leq 0.001$) as assessed by the Mann-Whitney U test are marked by superscript letters: ^{a} between non-expanded day 6 blastocysts and non-expanded day 7 blastocysts, ^{b} between non-expanded day 6 blastocysts and hatching day 7 blastocysts. ICM = inner cell mass; TB = trophoblast.
