# Supplementary material for: Cell Arrest and Cell Death in Mammalian Preimplantation Development: Lessons from the Bovine Model
Source: PLoS One. 2011 Jul 21;6(7):e22121. doi: 10.1371/journal.pone.0022121 (PMC3141016; doi:10.1371/journal.pone.0022121)
Supplement: Table S5 — Calibration curves/CT-values for quantitative Real-Time-PCR. (PDF) [file pone.0022121.s007.pdf]

Table S5. Calibration curves/CT-values for quantitative Real-Time-PCR.

Transcript	Detection range		
	Plasmid-DNA-standard		Samples
	CT	Copy number*	CT
18S rRNA	5 - 26	2×10^9 - 2×10^3	9 - 15
H2AFZ	20 - 32	2×10^6 - 2×10^2	23 - 32
CASP3	16 - 36	2×10^6 - 2×10^1	28 -36
BIRC4	15 - 32	2×10^6 - 2×10^1	25 - 32
BAX	15 - 33	2×10^6 - 2×10^1	26 - 33
BCL2	17 - 35	2×10^6 - 2×10^1	32 - 35
BCL2L1	16 - 35	2×10^6 - 2×10^1	27 - 35
CASP9	17 - 36	2×10^6 - 2×10^1	31 - 36
STAT3	15 - 34	2×10^6 - 2×10^1	23 - 29
CASP8	16 - 34	2×10^6 - 2×10^1	n.d.
FAS	15 - 33	4×10^6 - 4×10^1	31 - 33
FASLG	16 - 35	2×10^6 - 2×10^1	32 - 35

*measurement limit per reaction; CT = threshold cycle; n.d. = not detected.
